# Effect of Vacuum Heat Treatment on Surface Hydrophobicity and Chemical Composition of Moso Bamboo for Natural Fiber Composites

**DOI:** 10.3390/polym18111276

**Published:** 2026-05-22

**Authors:** Zilu Liang, Haiyun Jiang, Yimin Tan

**Affiliations:** School of Packaging and Materials Engineering, Hunan University of Technology, Zhuzhou 412007, China

**Keywords:** heat treatment, bamboo, chemical composition, surface properties

## Abstract

To enhance the interfacial bonding between bamboo and the polymer matrix in natural fiber composites (NFCs), vacuum heat treatment was applied to moso bamboo strips at temperatures ranging from 140 to 180 °C with holding times of 4 and 6 h. The effects of treatment conditions on the surface characteristics and chemical composition of bamboo were systematically investigated. Scanning electron microscopy (SEM), contact angle measurements, and Fourier transform infrared spectroscopy (FTIR) were employed to evaluate the changes in microstructure, surface wettability, and the main functional groups including α-cellulose, hemicellulose, and lignin. The results indicate that the severity of heat treatment (temperature–time combination) significantly influences the physicochemical properties of bamboo. Hemicellulose, which exhibited the lowest thermal stability, underwent pronounced degradation above 140 °C and showed the most substantial compositional variation. Although the relative contents of α-cellulose and lignin increased with increasing treatment severity, their absolute contents decreased. The vacuum environment was found to retard the degradation of α-cellulose to some extent. At 180 °C, severe disruption of the cell wall structure was observed, accompanied by the deformation and collapse of cell lumens. In addition, heat treatment increased the surface contact angle, indicating enhanced hydrophobicity, with temperature exerting a more pronounced effect than treatment time. FTIR analysis revealed a marked reduction in the intensity of the C=O stretching vibration of hemicellulose (~1730 cm^−1^) and the O–H stretching vibration (~3400 cm^−1^), while the aromatic structure of lignin remained relatively stable. Overall, vacuum heat treatment effectively enhanced the surface hydrophobicity of bamboo, providing a theoretical basis and technical support for the development of bamboo-reinforced natural fiber composites.

## 1. Introduction

Natural Fiber Composites (NFCs) are composed of natural fibers (e.g., hemp, bamboo, flax, jute, sisal, and coconut shell fibers) and polymer matrices (e.g., polypropylene, polylactic acid, and epoxy resin). In recent years, driven by increasing environmental awareness and the demand for sustainable development, NFCs have attracted extensive attention and achieved rapid development [1,2,3].

The application of NFCs in the packaging sector has expanded significantly due to their inherent advantages, including biodegradability, low density, and cost-effectiveness. These features enable NFCs to serve as promising alternatives to conventional non-degradable packaging materials such as plastics and foams, in line with current environmental requirements, which have extended to multiple areas, including transportation cushioning, food packaging, and retail packaging, accompanied by continuous improvements in technological maturity. Moreover, with continued technological advances, natural fiber composites are expected to play an increasingly important role as green materials in various industries [1,2,4,5,6,7].

As a reinforcement material, bamboo offers significant advantages in natural fiber composites. It is a fast-growing resource that typically reaches maturity within 3–5 years, exhibiting a growth rate much higher than that of most wood species. Bamboo fiber is a renewable resource that aligns with the principles of sustainable development. It is associated with relatively low carbon emissions during cultivation and processing, while its rapid growth enables substantial carbon dioxide uptake, thereby contributing to the mitigation of greenhouse gas emissions. Bamboo fiber exhibits high tensile strength and modulus, enabling effective reinforcement the mechanical properties of polymer matrices. Its specific strength (the ratio of strength to density) can exceed than that of some synthetic fibers (e.g., glass fiber), making it an ideal choice for lightweight and high-strength natural fiber composites [4,8,9,10,11,12,13,14].

Despite the advantages of bamboo as a reinforcement material in natural composites, it also has certain limitations and challenges. In particular, bamboo fibers are hydrophilic, while most polymer matrices are hydrophobic. This mismatch in hydrophilicity and hydrophobicity results in weak interfacial adhesion between bamboo fibers and polymer matrices, which impairs the overall performance of the composites. To improve interfacial adhesion, bamboo fibers usually require additional surface modification (e.g., alkali treatment, silane treatment, plasma treatment), which increases the complexity and cost of the manufacturing process [15,16,17,18,19].

Heat treatment is an effective approach that improves the properties of bamboo by controlling temperature and time. This heat treatment process features operational simplicity, causes little environmental pollution, and poses low risks to human health, thus having been widely applied in the field of wood modification [20,21,22]. Heat treatment methods mainly include oil heat treatment, hydrothermal treatment, and vapor phase heat treatment. Among these, the first two have been applied in small-scale industrialization worldwide due to their capacity to enhance the physical and chemical properties of bamboo. However, hydrothermal treatment and oil heat treatment feature complex processes and long processing times. Additionally, bamboo absorbs large amounts of oil or water during the treatment, which is unfavorable for subsequent processing and utilization [23,24]. Moreover, previous studies have shown that oxygen in the air accelerates the thermal degradation of bamboo. Although heat treatment can improve the surface properties of bamboo, it may also deteriorate its mechanical properties, leading to a rapid decline in its performance as a reinforcement material. Meanwhile, when the heat treatment temperature exceeds 200 °C, the mechanical properties of bamboo undergo a significant decrease [25,26]. This is unfavorable for its promotion and application as a reinforcement in natural fiber composites. Recent studies have further advanced the understanding of bamboo heat treatment. Chen et al. [27] systematically evaluated the carbon footprint of reconstituted bamboo under different heat treatment conditions, confirming the environmental benefits of thermal modification. Meanwhile, Li et al. [28] demonstrated that vacuum conditions significantly influence bamboo’s heat transfer characteristics and anti-mold performance. Zhang et al. [29] investigated the effect of thermal modification on the mechanical properties of laminated bamboo, revealing important trade-offs between hydrophobicity enhancement and mechanical performance. These findings collectively support the application of vacuum heat treatment as a promising strategy for bamboo modification. Therefore, this study adopts vacuum heat treatment technology to process bamboo, controlling the heat treatment temperature within the range of 140–180 °C, in order to investigate the effects of heat treatment on the properties of bamboo.

## 2. Materials and Methods

### 2.1. Materials

To investigate the effects of heat treatment on bamboo properties, 3–4-year-old moso bamboo (*Phyllostachys edulis*) harvested from Guangdong, China, was used. First, the green outer layer and yellow inner layer of the bamboo were removed, and the bamboo was cut into strips with dimensions of approximately 2 mm (thickness) × 10 mm (width) × 300 mm (length). The prepared bamboo strips were conditioned at 23 °C and 65% relative humidity for 15 days, followed by air-drying. Subsequently, the samples were planed and ground to obtain flat and smooth surfaces. The initial chemical composition of the untreated moso bamboo is as follows: α-cellulose ~46%, hemicellulose ~24%, lignin ~26%, ash ~1–2%, and extractives ~2–3% (based on previous studies on the same species [22,30]. This provides a baseline for comparing the changes induced by heat treatment. Moso bamboo (*Phyllostachys edulis*) was selected because it is the most widely cultivated and commercially important bamboo species in China, and its uniform properties make it a representative raw material for natural fiber composites research.

### 2.2. Heat Treatment of Bamboo

**Figure 1:**
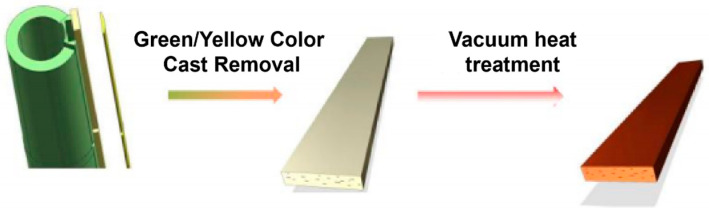
Bamboo processing and heat treatment.

The bamboo strips were arranged in a single layer with gaps between them to avoid stacking. The vacuum level was kept at approximately −0.1 MPa (relative pressure) continuously throughout the heating and holding stages. The temperature was increased from room temperature to the target temperatures (140, 160, and 180 °C). Upon reaching the desired temperature, the bamboo strips were held for 4 h or 6 h. After heat treatment, the samples were cooled to room temperature (approximately 23 °C) inside the oven. The preparation process of the bamboo samples is shown in Figure 1.

### 2.3. Contact Angle

According to the ASTM D7490-2013 standard [31], the contact angles of bamboo before and after heat treatment were measured using a contact angle measuring instrument (OCA 20, DataPhysics Instruments GmbH, Dusseldorf, Germany). Deionized water was used as the test liquid, and a droplet volume of 5 μL was applied for each measurement. Images of liquid droplets on the bamboo surface were captured, and the droplet profiles were analyzed using the instrument’s built-in image analysis software. The contact angles were then calculated based on the fitted droplet contours.

### 2.4. Microstructure

The cross-sectional microstructure of bamboo before and after heat treatment was characterized via a scanning electron microscope (SEM, Apreo 2S HiVac, Thermo Fisher Scientific, Waltham, MA, USA). In addition, the surface morphology of the samples was examined with a digital microscope at a magnification of 1600×.

### 2.5. FTIR

The chemical variations of functional groups on the bamboo surface before and after heat treatment were characterized using an attenuated total reflection Fourier transform infrared (ATR-FTIR) spectrometer (Bruker Tensor II, Bruker Optics GmbH, Ettlingen, Germany). The spectra were recorded in the wavenumber range of 800–4000 cm^−1^.

### 2.6. Chemical Composition

The contents of holocellulose and α-cellulose were determined by standard chemical methods. Subsequently, the content of hemicellulose was calculated by subtracting the content of α-cellulose from that of holocellulose [32].

#### 2.6.1. Determination of α-Cellulose Content

This experiment was conducted in accordance with GB/T 744-1989 (Determination of α-Cellulose in Pulp) [33]. The samples were treated with a 17.5% sodium hydroxide solution, then washed with a 9.5% sodium hydroxide solution and rinsed with deionized water. Subsequently, the samples were dried and weighed to quantitatively determine the α-cellulose content.

#### 2.6.2. Determination of Holocellulose

Holocellulose content was determined using the anthrone colorimetric method. Under acidic conditions and upon heating, cellulose is hydrolyzed to glucose. The resulting glucose then reacts with anthrone reagent in strong acid to produce a colored complex, the absorbance of which is measured to quantify the cellulose content.

#### 2.6.3. Determination of Lignin Content

The acetyl bromide method was adopted for the test. The phenolic hydroxyl groups in lignin were acetylated to form acetyl lignin, and the product exhibited a characteristic absorption peak at 280 nm. The lignin content was quantitatively determined according to the variation in absorbance value. In addition to the three major organic components described above, bamboo also contains inorganic minerals (ash). According to literature, moso bamboo typically contains about 1–2% ash, mainly composed of silicon (Si), potassium (K), calcium (Ca), and trace amounts of other elements [21].

## 3. Results and Discussion

### 3.1. Effects of Heat Treatment on the Microstructure of Bamboo

**Figure 2:**
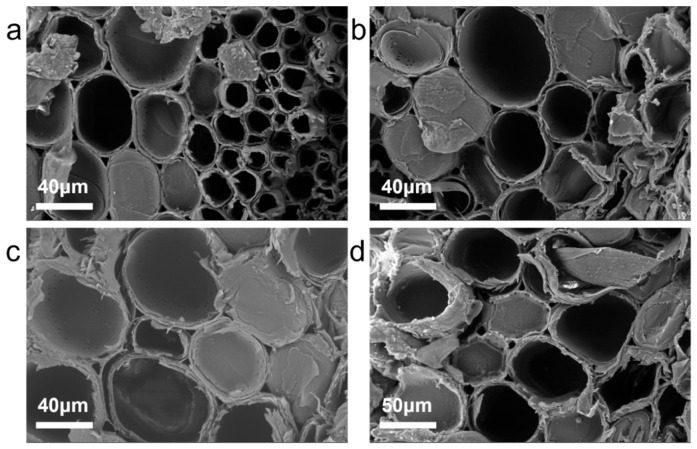
Cross-sectional SEM images of bamboo samples subjected to vacuum heat treatment at different temperatures: (**a**) untreated; (**b**) 140 °C for 4 h; (**c**) 160 °C for 4 h; (**d**) 180 °C for 4 h.

Figure 2 shows the cross-sectional SEM images of bamboo before and after vacuum heat treatment at different temperatures (140, 160, and 180 °C) for 4 h. For the untreated sample (Figure 2a), the cell wall structure in the cross-section was intact, with tight intercellular connections. After heat treatment at 140 °C (Figure 2b), the cell wall structure remained basically intact, with only minor changes observed, and the smoothness of the cell lumen walls was largely maintained. When being heat-treated at 160 °C for 4 h (Figure 2c), the bamboo exhibited obvious degradation of the cell wall structure; some cell lumens were slightly expanded due to the thinning of the cell walls, and a small amount of unevenness appeared on the lumen walls. When the temperature increased to 180 °C (Figure 2d), the cell wall structure was severely disrupted, accompanied by the enhanced degradation of cell wall components and pronounced morphological changes. The cell lumens exhibited significant deformation, and local ruptures were observed in the lumen walls.

### 3.2. Changes in Chemical Composition of Bamboo Induced by Heat Treatment

The microstructural changes induced by heat treatment are primarily associated with variations in the chemical composition of bamboo. Hemicellulose, with its unstable multi-branched and amorphous structure, exhibits poor thermal stability. At 160 °C and 180 °C, extensive degradation of hemicellulose reduces cell wall toughness and weakens cellulose microfibril connections, leading to progressive cell wall deterioration, manifested as increased cracking and wall thinning. Lignin softens and migrates slightly into cell wall cracks at 160 °C; at 180 °C, drastic softening and migration to the cell wall interior or surface form local aggregates, altering intercellular binding force and reducing adhesion [30,34].

**Figure 3:**
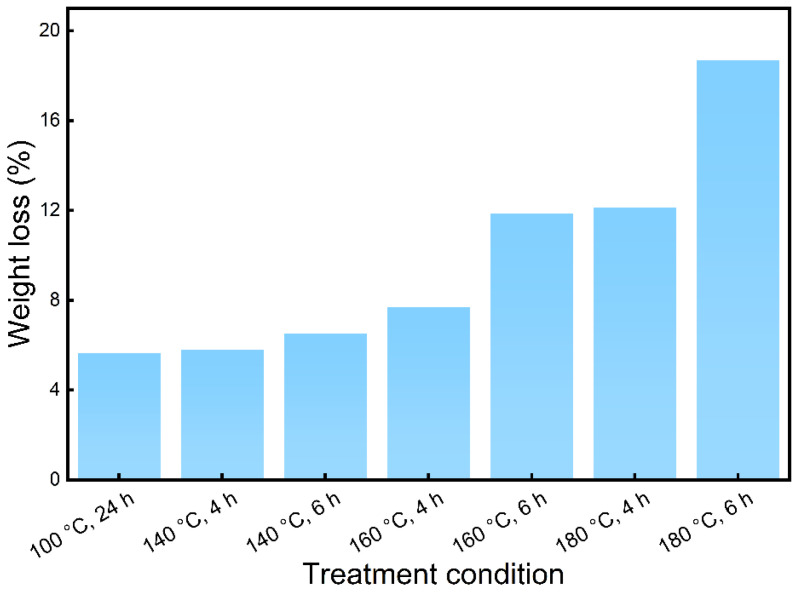
Weight loss of bamboo samples under different vacuum heat treatment conditions.

After heat treatment, the three main chemical components (cellulose, hemicellulose, lignin) changed significantly (Table 1). These changes are key to the improvement of bamboo properties (e.g., water absorption, dimensional stability). The sum of cellulose, hemicellulose, and lignin accounts for approximately 95% of the total dry mass; the remaining fraction is composed of ash and extractives. As shown in Table 1, volatile low-molecular-weight compounds in samples escape first at the initial heat treatment stage (100–140 °C). With increasing temperature and holding time (140–160 °C), thermally unstable components such as hemicellulose degrade, and cellulose and lignin gradually began to degrade at 160 °C, and further degraded at 180 °C. Thus, mass loss intensifies with increased heat treatment intensity (temperature–time combination) (Figure 3).

Chemical composition analysis reveals that bamboo’s main components decrease continuously with increasing heat treatment intensity: hemicellulose (69.03%), α-cellulose (28.67%), and lignin (19.51%) (Figure S1), consistent with findings by Lee [30] and Meng [22].

As shown in Figure S1, hemicellulose undergoes the most significant changes. With an amorphous, low-polymerization, branched structure and hydrophilic groups on main/side chains, it is the least thermally stable among main components, undergoing drastic degradation (dehydration, glycosidic bond cleavage) above 140 °C. Thus, its content drops sharply with increasing heat treatment intensity [35,36]. The absolute mass of hemicellulose decreased by 69.03% (calculated from Table 1) under the most severe treatment (180 °C, 6 h).

As shown in Table 1, α-cellulose content increases continuously with rising heat treatment intensity, while its absolute mass decreases consistently. With better thermal stability than hemicellulose, it shows no significant mass/content changes at 140 °C but undergoes molecular chain scission and dehydration at 160 °C and above, gradually degrading with increased heat treatment intensity [34]. The change in α-cellulose content differs from the results of some previous studies [22,30], which might be because under vacuum conditions, moisture and small-molecule compounds (e.g., acetic acid) from hemicellulose decomposition are removed, retarding α-cellulose degradation to a certain extent. Compared with the more severe α-cellulose degradation observed under ambient air heat treatment at similar temperatures [22,30], our vacuum-treated samples showed a smaller absolute loss, suggesting that the vacuum environment helps retard cellulose decomposition. Meanwhile, rapid hemicellulose degradation may also contribute to the increased α-cellulose content, which is conducive to enhancing bamboo’s mechanical properties [23].

Lignin is a complex aromatic polymer of phenylpropane units connected by C-C and C-O-C bonds, with thermal stability superior to hemicellulose and cellulose [37]. Lignin content shows a significant upward trend with the increase in heat treatment temperature and prolongation of heat treatment time, while its absolute mass continuously decreases. There are two main possible reasons for the increase in lignin content: first, lignin undergoes polycondensation reaction during heat treatment, where both its degree of polymerization and structure change, thereby forming a reticular cross-linked structure; second, the mass fraction of lignin increases due to the degradation of hemicellulose and cellulose [17,22,34,38].

### 3.3. FTIR Spectroscopic Characteristics and Analysis of Bamboo Before and After Heat Treatment

**Figure 4:**
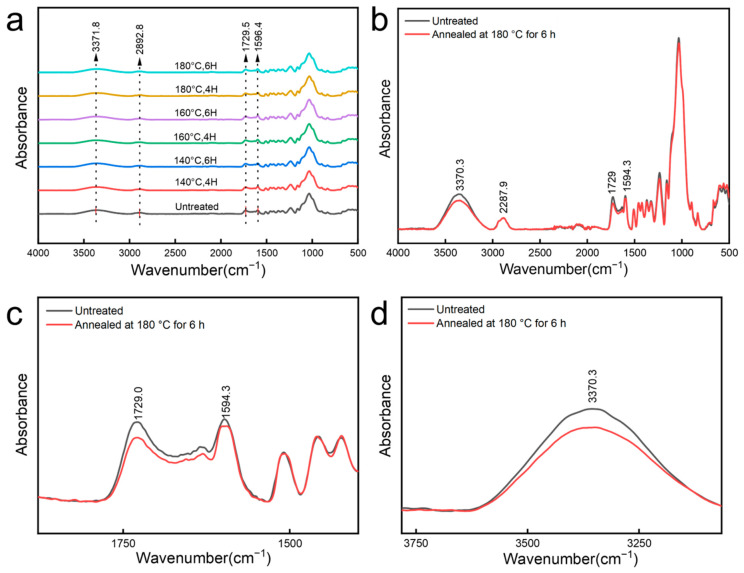
FTIR spectra of bamboo under different heat treatment conditions. (**a**) FTIR spectra of bamboo under different heat treatment conditions; (**b**) comparison between untreated and heat-treated (180 °C, 6 h) samples; (**c**) enlarged view of the region 1800–1400 cm^−1^; (**d**) enlarged view of the O–H stretching region (3700–3000 cm^−1^).

As an important method to improve bamboo properties, heat treatment, combined with FTIR analysis, provides insight into the changes in the main chemical components of bamboo (cellulose, hemicellulose, and lignin) during heat treatment [21,22,23,24,39]. Figure 4 illustrates the variations in the characteristic peaks of the FTIR spectra of bamboo before and after heat treatment.

Among the characteristic absorption peaks of bamboo, the C-H stretching vibration peak of methyl and methylene groups in cellulose is located at 2890 cm^−1^. Owing to the high bond energy of C-H bonds in the cellulose main chain, heat treatment below 200 °C only induces slight molecular thermal vibration without damaging the number or vibration characteristics of chemical bonds. As a result, the peak position remains stable, the intensity fluctuation is small, and no significant attenuation is observed. Thus, this peak can serve as a reference for calculating relative values (target peak area/reference peak area), thereby offsetting external systematic errors and enhancing the comparability of target peaks (e.g., hemicellulose: 1729 cm^−1^; lignin: 1594 cm^−1^) (Table 2).

The characteristic peak near 1590 cm^−1^ corresponds to the C=C stretching vibration absorption peak of the aromatic ring in lignin [10,40,41]. After heat treatment, the change in the intensity of the absorption peak near 1590 cm^−1^ is relatively small. The core structure of the aromatic ring in lignin has high bond energy and does not break below 200 °C, but high temperatures cause slight detachment of the side chains (e.g., methoxy groups) of the aromatic ring, which indirectly affects the conjugated system of the aromatic ring and thus leads to a gradual decrease in peak intensity [22,24]. The stability of the aromatic ring increases its relative proportion, while its absolute content decreases slightly. Compared with that before treatment, the maximum reduction in the characteristic peak area is approximately 7%. It should be noted that FTIR peak intensity represents the absolute abundance of the corresponding functional groups. Although the relative lignin content increases after heat treatment due to the preferential degradation of hemicellulose and cellulose, its absolute mass decreases, leading to a reduction in the 1597 cm^−1^ peak intensity.

The characteristic peak near 1730 cm^−1^ is assigned to the C=O stretching vibration of hemicellulose, arising from acetyl and carboxyl groups [40]. Post-heat treatment, the relative area of this C=O peak drops significantly, demonstrating hemicellulose degradation at elevated temperatures (Hemicellulose is rich in acetyl and uronic acid units; C=O bond cleavage and deacetylation are key degradation processes, causing attenuation or disappearance of the 1730 cm^−1^ peak [10,23,24,35]). Hemicellulose has poor stability, and the change in the relative area of the absorption peak at 1728 cm^−1^ further confirms its degradation. Compared with that before treatment, the maximum reduction in the absorption peak area was approximately 45%.

The characteristic peak near 3400 cm^−1^ corresponds to the O–H stretching vibration absorption peak of cellulose/hemicellulose [23,42,43]. After heat treatment, the intensity of the O–H stretching vibration peak (3400 cm^−1^) decreases. This is because heat treatment removes bound water in bamboo, reducing the number of free hydroxyl groups (-OH). Meanwhile, the crystallinity of cellulose increases and the amorphous regions decrease, thus leading to a reduction in the intensity of the O–H stretching vibration peak [23,44]. As discussed by Geminiani et al. [45], higher spectral resolution (≤2 cm^−1^) and band deconvolution are required to resolve the overlapping O–H stretching bands of cellulosic materials. Zhang et al. [21] used second-derivative FTIR spectroscopy to resolve the O–H stretching region of heat-treated bamboo, attributing the decrease in broad O–H band intensity to a reduction in free hydroxyl groups and enhanced intra-molecular hydrogen bonding due to cellulose recrystallization. Hu et al. [20] applied vacuum heat treatment to bamboo fibers and observed a similar decrease in O–H stretching intensity and increase in hydrophobicity, which is consistent with our findings. The structure of cellulose is relatively stable, which is mainly manifested as dehydration and increased crystallinity, and its degradation degree is lower than that of hemicellulose. Compared with that before treatment, the maximum reduction in the area of the characteristic absorption peak was approximately 22%.

As can be seen from the comparison of the change rates of the relative areas of characteristic peaks, when the heat treatment temperature is below 160 °C, the changes in the characteristic peaks are relatively small. When the temperature increases to 180 °C, the relative areas of the characteristic peaks undergo significant changes, and the influence of temperature on the changes in the relative areas of characteristic peaks is greater than that of time.

### 3.4. Changes in Contact Angle of Bamboo Before and After Heat Treatment

**Figure 5:**
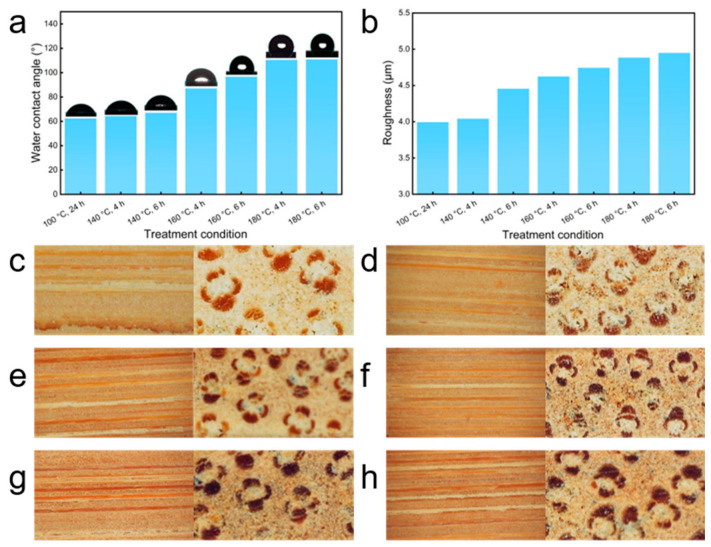
Changes in surface wettability, roughness, and microstructure of bamboo subjected to different heat treatment conditions: (**a**) Water contact angle; (**b**) Surface roughness (Ra); (**c**–**h**) Surface (left) and cross-sectional (right): (**c**) 140 °C, 4 h; (**d**) 140 °C, 6 h; (**e**) 160 °C, 4 h; (**f**) 160 °C, 6 h; (**g**) 180 °C, 4 h; (**h**) 180 °C, 6 h.

After heat treatment, the surface contact angle of bamboo tends to change, which reflects the variation in the surface wettability of bamboo, as shown in Figure 5. The surface contact angle of bamboo increases, indicating a decrease in its surface wettability and an enhancement in hydrophobicity. Under low-temperature treatment conditions (e.g., below 160 °C), the contact angle increases, but the change is relatively small. In contrast, under a high temperature of 180 °C, the contact angle increases significantly.

Heat treatment modifies the chemical composition and microstructure of the bamboo surface, thereby exerting an impact on its wettability. Hemicellulose is rich in a large amount of hydrophilic groups (e.g., hydroxyl and carboxyl groups); its degradation after heat treatment leads to a reduction in these hydrophilic groups, resulting in an increase in contact angle. Meanwhile, lignin starts to flow when the temperature exceeds 160 °C, which further contributes to the enhancement of hydrophobicity. In addition, when upon heating, the fatty substances and waxes in bamboo may migrate to the surface layer, forming a water-repellent film and thus enhancing the hydrophobicity of bamboo. As the temperature increases further, the fatty substances and waxes degrade and release volatile products, which further affect the hydrophobicity of the bamboo surface [20,30].

Heat treatment not only modifies the chemical composition of bamboo, but also induces changes in the microstructure of bamboo during the treatment process, leading to an increase in bamboo surface roughness, as shown in Figure 5. Changes in roughness exert a significant influence on the wetting behavior of liquids on solid surfaces, thereby altering the contact angle. When the roughness is low (Ra < 5 μm), the changes in roughness and contact angle are dominated by the Wenzel model, as defined in Equation (1):

*cos θ_r_* = *rcos θ*_0_ (1)

where *θ_r_* is the contact angle of the rough surface, r is the surface roughness factor, and *θ*_0_ is the contact angle of the smooth surface. It should be noted that the surface roughness factor was not directly measured; the arithmetic average roughness *R_a_* was used as a qualitative indicator. The Wenzel model is employed here only to explain the observed trend of contact angle changes with increasing roughness.

When the surface contact angle of bamboo is less than 90°, the surface is relatively hydrophilic. At this time, the increase in surface roughness induced by heat treatment provides more attachment sites for liquids, making it easier for liquids to spread out on the surface and thus reducing the contact angle. With the continuous increase in heat treatment intensity, when the contact angle exceeds 90°, the bamboo surface itself is hydrophobic. The increased roughness leads to more protrusions on the surface, making it more difficult for liquids to overcome these barriers and spread, which consequently increases the contact angle and causes the deterioration of wettability. For bamboo surfaces with contact angles exceeding 90° after heat treatment at 180 °C, a transition from the Wenzel regime to the Cassie–Baxter regime may occur, where air pockets trapped beneath the liquid droplet further enhance surface hydrophobicity. This potential mechanism is consistent with the observed significant increase in contact angle at 180 °C.

Factors such as the coverage of lignin/water-repellent film (fatty substances and waxes) and the reduction in hemicellulose lead to a decrease in polar groups. Meanwhile, the destruction of the cell wall microstructure of bamboo results in the formation of a porous structure and an increase in surface roughness. The roughening of the hydrophobic solid surface, together with these structural changes, jointly promotes an increase in contact angle and a significant decrease in wettability. Furthermore, the influence of temperature on the contact angle is greater than that of time during the heat treatment process.

Although the present study does not include composite fabrication, the observed increase in surface contact angle and reduction in hydrophilic groups (e.g., O–H and C=O) suggest that vacuum heat-treated bamboo should exhibit improved interfacial adhesion with hydrophobic polymer matrices such as PP or PLA. Based on previous reports on heat-treated plant fibers [20,30], the enhanced hydrophobicity is expected to reduce water absorption at the interface and delay the initiation of debonding. Future studies will focus on preparing bamboo fiber reinforced polymer composites to directly verify these hypotheses, including measurements of interfacial shear strength, tensile/flexural properties, and water resistance.

## 4. Conclusions

In this study, we investigated the effects of vacuum heat treatment on moso bamboo at 140–180 °C for 4 and 6 h. Hemicellulose, the least thermally stable component, degraded most severely, with its absolute mass decreasing by up to 69.03% at 180 °C for 6 h. The vacuum environment retarded α-cellulose degradation; although the absolute masses of α-cellulose and lignin decreased, their relative contents increased due to preferential hemicellulose loss. Bamboo surface hydrophobicity increased significantly after heat treatment, especially at 180 °C, as indicated by a higher water contact angle. FTIR analysis confirmed a marked reduction in the C=O peak (~1730 cm^−1^) and O–H peak (~3400 cm^−1^), while lignin’s aromatic structure remained stable. Severe cell wall disruption, including lumen deformation and collapse, was observed at 180 °C. Overall, vacuum heat treatment effectively improves bamboo surface hydrophobicity and may enhance interfacial compatibility with hydrophobic polymer matrices. Future work should fabricate bamboo-reinforced composites to directly verify these benefits.

## Figures and Tables

**Table 1:** Mass changes and chemical composition of bamboo before and after vacuum heat treatment.

Sample Group	Initial Mass/g	Final Mass/g	Holocellulose/%	α-Cellulose/%	Hemicellulose/%	Lignin/%
100 °C, 24 h	10.14	9.61	69.89	46.07	23.82	25.96
140 °C, 4 h	11.73	11.05	69.21	47.91	21.30	25.76
140 °C, 6 h	10.38	9.27	67.41	47.02	20.39	27.59
160 °C, 4 h	9.66	8.26	66.13	48.54	17.59	29.36
160 °C, 6 h	9.86	7.94	64.47	50.37	14.11	29.95
180 °C,4 h	9.99	7.54	62.28	50.80	11.49	31.82
180 °C,6 h	10.02	7.39	63.30	51.62	11.68	31.75

**Table 2:** Relative changes in FTIR peak intensity ratios of bamboo under different heat treatment conditions.

Sample Group	Reference Peak (2890 cm^−1^)	Target Peak (1728 cm^−1^)		Target Peak (1597 cm^−1^)		Target Peak (3400 cm^−1^)	
	Peak Area (Abs cm^−1^)	Relative Area	Change Rate	Relative Area	Change Rate	Relative Area	Change Rate
Untreated	0.49	2.54	0.00%	1.77	0.00%	7.92	0.00%
140 °C, 4 h	0.49	2.43	−4.52%	1.79	1.15%	7.41	−6.44%
140 °C, 6 h	0.48	2.41	−5.24%	1.78	0.72%	7.30	−7.80%
160 °C, 4 h	0.49	2.18	−14.18%	1.71	−3.99%	6.62	−16.40%
160 °C, 6 h	0.49	1.56	−38.55%	1.71	−4.13%	6.55	−17.27%
180 °C, 4 h	0.49	1.39	−45.44%	1.60	−6.10%	6.50	−17.92%
180 °C, 6 h	0.48	1.33	−47.60%	1.59	−6.89%	6.21	−21.60%

## Data Availability

The original contributions presented in this study are included in the article/Supplementary Material. Further inquiries can be directed to the corresponding authors.

## Supplementary Materials

The following supporting information can be downloaded at: https://www.mdpi.com/article/10.3390/polym18111276/s1, Figure S1: Relative changes in bamboo chemical components under different heat treatment conditions.
