# Supplementary material for: Effect of Vacuum Heat Treatment on Surface Hydrophobicity and Chemical Composition of Moso Bamboo for Natural Fiber Composites
Source: Polymers (Basel). 2026 May 22;18(11):1276. doi: 10.3390/polym18111276 (PMC13259473; doi:10.3390/polym18111276)
Supplement: Supplementary file 1 [file polymers-18-01276-s001.zip › polymers-4316113-supplementary.pdf]

# Supporting Information

# Effect of Vacuum Heat Treatment on Surface Hydrophobicity and Chemical Composition of Moso Bamboo for Natural Fiber Composites

**Zilu Liang*, Haiyun Jiang, and Yimin Tan***

School of Packaging and Materials Engineering, Hunan University of Technology, Zhuzhou, 412007, China

* Correspondence: Corresponding author: Zilu Liang, e-mail: lzl98qindao@126.com

Yimin Tan, e-mail: csfutanyimin@126.com:

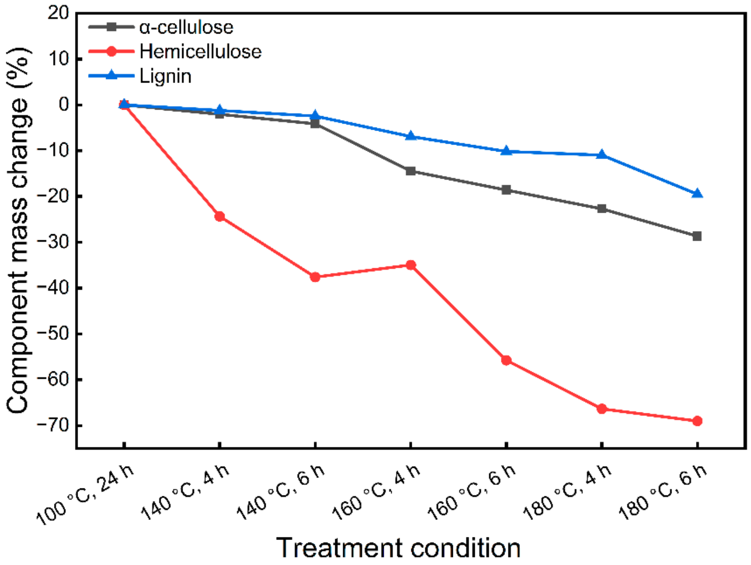


**Figure S1.** Relative changes in bamboo chemical components under different heat treatment conditions.
